# Association between *PPARγ*, *PPARGC1A*, and *PPARGC1B* genetic variants and susceptibility of gastric cancer in an Eastern Chinese population

**DOI:** 10.1186/s12920-022-01428-0

**Published:** 2022-12-31

**Authors:** Boyang Chen, Yafeng Wang, Weifeng Tang, Yu Chen, Chao Liu, Mingqiang Kang, Jinbiao Xie

**Affiliations:** 1 Department of Cardiothoracic Surgery, The Affiliated Hospital of Putian University, Putian, 351100 Fujian Province China; 2 Department of Cardiology, The People’s Hospital of Xishuangbanna Dai Autonomous Prefecture, Jinghong, Yunnan Province China; 3 Department of Cardiothoracic Surgery, Nanjing Drum Tower Hospital, The Affiliated Hospital of Nanjing University Medical School, Nanjing, Jiangsu Province China; 4 Department of Medical Oncology, Fujian Cancer Hospital, Fujian Medical University Cancer Hospital, Fuzhou, Fujian Province China; 5 Department of Cardiothoracic Surgery, Affiliated People’s Hospital of Jiangsu University, Zhenjiang, Jiangsu Province China; 6 Department of Thoracic Surgery, Fujian Medical University Union Hospital, Fuzhou, 350001 Fujian Province China

**Keywords:** *PPARγ*, *PPARGC1A*, *PPARGC1B*, Variant, SNVs, Gastric cancer

## Abstract

**Purpose:**

Previous studies showed that peroxisome proliferator-activated receptor gamma (*PPARγ*) and *PPARγ* coactivator1 family (*PPARGC1A* and *PPARGC1B*) gene single nucleotide variants (SNVs)were strongly associated with cancer susceptibility. The purpose of this study was to investigate the association of *PPARγ*, *PPARGC1A*, and *PPARGC1B* variants with the risk of gastric cancer (GC).

**Patients and methods:**

We performed a case-control study of 490 GC cases and 1,476 healthy controls from eastern China. *PPARγ* rs1801282 C > G, rs3856806 C > T, *PPARGC1A* rs2970847 C > T, rs8192678 C > T and *PPARGC1B* rs7732671 G > C, rs17572019 G > A SNVs were selected to investigate the association between these SNVs and GC susceptibility. Genotypes of the SNVs were assessed by multiplex fluorescent PCR using a custom-by-design 48-Plex SNPscan^tm^ Kit.

**Results:**

The *PPARγ* rs1801282 SNV was associated with a decreased risk for GC (GC vs. CC: odds ratio (OR) = 0.62, 95% confidence interval (95%CI) = 0.42–0.93, adjusted *P* = 0.019; GC + GG vs. GG: OR = 0.63 95%CI = 0.42–0.93, adjusted *P* = 0.019; respectively). In addition, stratified analysis revealed that the *PPARγ* rs1801282 SNV was correlated with the risk of GC in subgroups of age ≥ 61, no smoking, and no alcohol consuming. We also confirmed that the *PPARγ* rs3856806 C > T SNV promoted the risk of GC in women. The *PPARGC1A* rs8192678 TT genotype decreased the susceptibility of GC in men. The *PPARGC1A* rs2970847 C > T SNV decreased the susceptibility of GC in the subgroup of BMI ≥ 24 kg/m^2^. The *PPARGC1B* rs7732671 G > C and rs17572019 G > A SNVs promoted the risk of GC in the subgroup of BMI ≥ 24 kg/m^2^.

**Conclusion:**

This study indicates that the *PPARγ*, *PPARGC1A*, and *PPARGC1B* SNVs may be associated with the susceptibility of GC in eastern Chinese population. Future studies with larger populations, detailed *H. pylori* infection status for subgroup analysis, and functional study are needed to further clarify the relationship between these SNVs and GC risk.

**Supplementary Information:**

The online version contains supplementary material available at 10.1186/s12920-022-01428-0.

## Introduction

Gastric cancer (GC) accounts for more than 5% of all new cancer cases worldwide, making it the fifth most common cancer and the third leading cause of cancer-related deaths [1]. China is the worst affected, accounting for 45% of all cancer-related deaths and 42.6% of the incidence [2]. Notably, GC is often diagnosed at an advanced stage due to the lack of effective diagnostic markers, leading to a poor prognosis with a 5-year survival rate of below 40% [3]. Various risk factors influence the incidence of GC, such as microbial infections, genetic factors, alcohol, dietary regime, and obesity [4]. Many hereditary factors were found to play an impact on susceptibility to GC.

*Peroxisome activated receptor gamma* (*PPARγ*), a type II nuclear receptor gene, located on chromosome 3p25, is a member of the peroxisome activated receptor (PPAR) superfamily involved in adipogenesis, lipid metabolism, cell proliferation, chronic inflammation, and insulin sensitivity [5]. The aberrant *PPARγ* signaling pathway was associated with the development of obesity, diabetes, and cancers [6]. PPAR is overexpressed in various malignant tissues, including breast, esophageal, gastrointestinal, and prostate cancers [7]. Several studies suggested that *PPARγ* expression was associated with the prognosis of various tumors, including cancers of the breast, pancreas, and colorectum [8–10]. When activated by ligands, PPAR can act as a tumor suppressor by inducing tumor cell differentiation, inhibiting proliferation, promoting apoptosis, and reducing tumorigenic angiogenesis [11, 12]. PPAR receptor agonists exert inhibitory effects on various types of tumor cells and exert synergistic effects on chemotherapy and radiotherapy [13–15]. PPARGC1A and PPARGC1B are transcriptional coactivators of the PPAR superfamily, which share a high sequence identity [16–18]. They are well-established as master regulators of oxidative phosphorylation and fatty acid oxidation gene expression and are highly expressed in oxidative tissues such as brown adipose tissue, heart, kidney, skeletal muscle, and brain [19, 20]. The PPARGC1 family has been reported to play an important role in cancer progression by promoting the expression of antioxidant genes, regulating the expression of vascular endothelial growth factor, and promoting glucose metabolism and adipogenesis [21–23]. Increased expression and activity of PPARGC1A in cancers of lung, prostate, cervical, breast, colon, and melanoma promoted cancer cell progression and chemoresistance [21, 24]. However, PPARGC1A expression was significantly lower in clear cell renal cell cancer tissues and associated with a favorable prognosis [25, 26]. PPARGC1B enhances the mitochondrial activity and anabolic profile, contributing to the development of hepatocellular and intestinal cancers [27, 28]. Estrogen-related receptor α (ERRα) was highly expressed in GC tissues and promoted cancer progression[29]. PPARGC1A, and PPARGC1B, are also co-activators of ERRα, which could be related to the development of GC.

Single nucleotide variant (SNV) is the most common type of genetic variant that affects gene expression through different mechanisms and is associated with genetic susceptibility to cancer [30]. In the previous studies, we compared the *PPARγ*, *PPARGC1A*, and *PPARGC1B* SNVs with susceptibility of esophageal, colorectal, and hepatocellular carcinomas [31–33]. Therefore, we assumed that *PPARγ*, *PPARGC1A*, and *PPARGC1B* SNVs might be effective susceptibility markers of GC. According to the previous studies [31–35], *PPARγ* rs1801282 C > G, rs3856806 C > T, *PPARGC1A* rs8192678 C > T rs2970847 C > T, and *PPARGC1B* rs7732671 G > C, rs17572019 G > A SNVs are chosen to investigate the association between these SNVs and GC risk in a Chinese cohort.

## Materials and methods

This cohort is part of a previous study [35]. GC group were recruited from in patients of the Affiliated People’s Hospital of Jiangsu University (Zhenjiang, Jiangsu province, China) and the Affiliated Union Hospital of Fujian Medical University (Fuzhou, Fujian Province, China). Healthy controls were recruited from participants in health screening at the same hospitals. Only Han Chinese population of East China residents without autoimmune diseases or other malignancies were included. The main inclusion criteria for GC cases were sporadic newly diagnosed primary GC patients with pathological confirmation; while GC cases who underwent chemotherapy prior to blood sample collection were excluded from the cohort. Healthy controls were age and gender-matched volunteers who underwent health checkups and were excluded from autoimmune diseases or malignancies. Demographic information and correlated risk factor were obtained through medical records and supplemental interviews. All participants enrolled were voluntary and signed an informed consent document in advance. The protocol was approved by the institutional ethics committees of Fujian Medical University (No.K201433).

### DNA extraction and genotyping

In this study, 2mlEDTA anticoagulant fasting peripheral venous blood was donated by each participant and stored at -70℃ until DNA extraction was performed. Genomic DNA extraction was conducted by using a commercial blood DNA extraction kit (Promega, Madison, USA) following the procedure according to the manufacturer’s instructions.

Simply put, cryopreserved blood samples were water bath thawing. After erythrocyte lysis and removal, nuclear release, protein precipitation, and removal, genomic DNA precipitation, and re-dissolve, genomic DNA was obtained. The concentration and purity of DNA were assayed for quality control by a microspectrophotometer.

As with the prior, the genotypes of the six SNVs were assessed by multiplex fluorescent PCR using a custom-by-design 48-Plex SNPscan^tm^ Kit (Genesky Biotechnologies Inc., Shanghai, China) [34]. For quality control, seventy-nine samples (4%) were randomly drawn and retested. No alteration in the result of the genotype was found.

The genotype frequency of each SNV in the control group was tested for deviation from Hardy-Weinberg equilibrium (HWE) by Pearson’s goodness-of-fit chi-square using online Chi-square software (http://ihg.gsf.de/cgi-bin/hw/hwa1.pl). Genotype frequencies of the SNVs variants were compared using a Chi-square test (χ2) or Fisher’s exact test among GC cases and controls. The relationship of the SNVs with susceptibility to GC was assessed by Multivariate logistic regression analysis to estimate by odds ratios (ORs) and 95% confidence intervals (CIs). The student’s t-test and chi-square (χ2) test were used to compare continuous variables and discrete variables between CG patients and healthy controls, respectively. A *P* < 0.05 (two-tailed) was considered statistically significant. All statistical analyses performed in the present study were conducted using the SPSS software package (SPSS, Inc., version 19.0, Chicago, Illinois, USA).

## Result

### Clinical characteristics

The Demographic variables and risk factors of the cohort are summarized in Table 1. A total of 1,966 subjects (490 GC cases and 1,476 health controls) were recruited for our study. No significant differences were found between GC patients and healthy controls in terms of gender and age ( Age: *P* = 0.026, Sex: *P* = 0.891). Thus, GC cases and controls were well matched. However, there was a significant higher in tobacco smoking, alcohol consumption, and body mass index (BMI) < 24 (kg/m2) in GC cases than in healthy controls (Alcohol use: *P* < 0.001, Smoking status: *P* = 0.001, BMI: *P* < 0.001 ).

**Table 1:** Distribution of selected demographic variables and risk factors in GC patients and controls

Variable	Overall cases (n = 490)	Overall controls (n = 1476)	*P*^a^
	N (%)	N (%)	
Age (years)	60.65 ± 11.43	61.30 ± 9.60	0.220
Age (years)			0.597
≥ 61	269 (54.90)	790 (53.52)	
< 61	221 (45.10)	686 (46.48)	
Sex			0.891
Male	331 (67.55)	1002 (67.89)	
Female	159 (32.45)	474 (32.11)	
Alcohol use			**< 0.001**
Never	374 (76.33)	1319 (89.36)	
Ever	116 (23.67)	157 (10.64)	
Smoking status			**0.001**
Never	309 (63.06)	1051 (71.21)	
Ever	181 (36.94)	425 (28.79)	
BMI (kg/m^2^)	22.41 ± 3.12	23.95 ± 3.05	**< 0.001**
BMI (kg/m^2^)			
< 24	356 (72.65)	761 (51.56)	**< 0.001**
≥ 24	134 (27.35)	715 (48.44)	

Bold indicates statistical significance (*P* < 0.05)

^a^Two-sided *χ*^2^ test and Student t test

### Data quality

As summarized in Table 2, the MAF values (minor allele frequency) of our controls were similar to the values for Chinese in the database.

**Table 2:** Primary information for *PPARγ* rs1801282 C > G, rs3856806 C > T, *PPARGC1A* rs8192678 C > T, rs2970847 C > T, *PPARGC1B* rs7732671 G > C, rs17572019 G > A polymorphisms

Genotyped SNPs	*PPARγ* rs1801282C > G	*PPARγ* rs3856806C > T	*PPARGC1A* rs8192678 C > T	*PPARGC1A* rs2970847 C > T	*PPARGC1B* rs7732671 G > C	*PPARGC1B* rs17572019 G > A
Chromosome: position	chr3: 12,393,125	chr3: 12,475,557	chr4: 23,815,662	chr4: 23,815,924	chr5: 149,212,243	chr5: 149,212,471
Function	Missense	Coding-synonymous	Missense	Coding-synonymous	Missense	Missense
Regulome DB score^a^	–	2b	6	–	5	5
Clinical significance^e^	Likely-benign	Benign/likely-benign	–	–	Benign	–
MAF^b^ for Chinese in database	0.07	0.25	0.35	0.28	0.09	0.07
MAF in our controls (n = 1476)	0.05	0.22	0.44	0.22	0.06	0.06
*P* value for HWE^c^ test in our controls	0.881	**0.026**^d^	0.954	0.492	0.497	0.139
Genotyping method	SNPscan	SNPscan	SNPscan	SNPscan	SNPscan	SNPscan
% Genotyping value	99.64%	99.64%	99.64%	99.64%	99.64%	99.53%

Bold indicates statistical significance (*P* < 0.05)

^a^https://www.regulomedb.org/.

^b^MAF: minor allele frequency

^c^HWE: Hardy–Weinberg equilibrium

^d^The genotype distribution of rs3856806 variant did not reach HWE

^e^https://www.ncbi.nlm.nih.gov/snp/

Five *P* values of the SNVs rs1801282, rs3856806, rs8192678, rs2970847, rs7732671, and rs17572019) from Hardy–Weinberg equilibrium (HWE) test were more than 0.05 (*P* = 0.881, 0.954, 0.492, 0.497, 0.139 respectively). However, the genotype distribution of rs3856806 variants did not reach HWE (*P* = 0.026).

Overall, all genotyping successful rates for the SNVs were > 95%, suggesting that the study met the requirements of molecular epidemiology.

### Association of PPARγ, PPARGC1A, and PPARGC1B SNVs with risk of GC

The distribution of genotypes and genotype frequencies are summarized in Table 3. We found that both in the additive and dominant model, the *PPARγ* rs1801282 SNV was associated with a decreased risk for GC (additive model: GC vs. CC: *P* = 0.033; dominant model: GC + GG vs. GG: *P* = 0.032; respectively). After adjusting for age, gender, BMI, smoking status, and alcohol consumption, the results remained statistically significant (GC vs. CC: adjusted *P* = 0.019; GC + GG vs. GG: adjusted *P* = 0.019; respectively). However, no significant relationship was found between GC risk and other genotypes assayed in genetic models (*PPARγ* rs3856806 C > T; *PPARGC1A* rs2970847 C > T; *PPARGC1A* rs8192678 C > T; *PPARGC1B* rs17572019 and *PPARGC1B*rs7732671).

**Table 3:** Logistic regression analyses of associations between *PPARγ* rs1801282 C > G, rs3856806 C > T, *PPARGC1A* rs8192678 C > T, rs2970847 C > T, *PPARGC1B* rs7732671 G > C, rs17572019 G > A polymorphisms and risk of GC

Genotype	Cases (n = 490)		Controls (n = 1476)		Crude OR (95%CI)	*P*	Adjusted OR^a^(95%CI)	*P*
*PPARγ r*s1801282 C > G								
CC	452	92.24	1317	89.23	1			
GC	34	6.94	151	10.23	0.66 (0.45–0.97)	**0.033**	0.62 (0.42–0.93)	**0.019**
GG	1	0.2	4	0.27	0.73 (0.08–6.53)	0.777	0.83 (0.08–8.25)	0.874
GC + GG	35	7.14	155	10.5	0.66 (0.45–0.96)	**0.032**	0.63 (0.42–0.93)	**0.019**
CC + GC	486	99.18	1468	99.46	1			
GG	1	0.2	4	0.27	0.76 (0.08–6.77)	0.802	0.86 (0.09–8.59)	0.9
G allele	36	3.67	159	5.39				
*PPARγ* rs3856806 C > T								
CC	278	56.73	868	58.81	1			
CT	188	38.37	544	36.86	1.08 (0.87–1.34)	0.486	1.08 (0.87–1.35)	0.482
TT	21	4.29	60	4.07	1.09 (0.65–1.83)	0.736	1.02 (0.60–1.74)	0.931
CT + TT	209	42.65	604	40.92	1.08 (0.88–1.33)	0.465	1.08 (0.87–1.33)	0.501
CC + CT	466	95.1	1412	95.66	1			
TT	21	4.29	60	4.07	1.06 (0.64–1.76)	0.818	0.99 (0.59–1.68)	0.977
T allele	230	23.47	664	22.49				
*PPARGC1A* rs8192678 C > T								
CC	169	34.49	454	30.76	1			
CT	236	48.16	726	49.19	0.87 (0.69–1.10)	0.248	0.89 (0.70–1.13)	0.346
TT	82	16.73	292	19.78	0.75 (0.56–1.02)	0.067	0.78 (0.57–1.07)	0.121
CT + TT	318	64.9	1018	68.97	0.84 (0.68–1.04)	0.113	0.86 (0.69–1.08)	0.189
CC + CT	405	82.65	1180	79.95	1			
TT	82	16.73	292	19.78	0.82 (0.63–1.07)	0.145	0.84 (0.63–1.11)	0.209
T allele	400	40.82	1310	44.38				
*PPARGC1A*rs2970847 C > T								
CC	303	61.84	890	60.3	1			
CT	160	32.65	515	34.89	0.91 (0.73–1.14)	0.415	0.90 (0.72–1.14)	0.384
TT	24	4.9	67	4.54	1.05 (0.65–1.71)	0.837	1.16 (0.70–1.91)	0.567
CT + TT	184	37.55	582	39.43	0.93 (0.75–1.15)	0.491	0.93 (0.75–1.16)	0.521
CC + CT	463	94.49	1405	95.19	1			
TT	24	4.9	67	4.54	1.09 (0.67–1.75)	0.732	1.20 (0.73–1.97)	0.471
T allele	208	21.22	649	21.99				
*PPARGC1B* rs7732671 G > C								
GG	436	88.98	1299	88.01	1			
GC	50	10.2	166	11.25	0.90 (0.64–1.25)	0.526	0.96 (0.68–1.36)	0.821
CC	1	0.2	7	0.47	0.43 (0.05–3.47)	0.425	0.42 (0.05–3.47)	0.42
GC + CC	51	10.41	173	11.72	0.88 (0.63–1.22)	0.442	0.94 (0.67–1.32)	0.708
GG + GC	486	99.18	1465	99.25	1			
CC	1	0.2	7	0.47	0.43 (0.05–3.51)	0.431	0.42 (0.05–3.48)	0.423
C allele	52	5.31	180	6.1				
*PPARGC1B* rs17572019 G > A								
GG	435	88.78	1298	87.94	1			
GA	50	10.2	165	11.18	0.90 (0.65–1.26)	0.555	0.98 (0.70–1.39)	0.916
AA	1	0.2	9	0.61	0.33 (0.04–2.63)	0.296	0.27 (0.03–2.21)	0.224
GA + AA	51	10.41	174	11.79	0.88 (0.63–1.22)	0.427	0.94 (0.67–1.32)	0.706
GG + GA	485	98.98	1463	99.12	1			
AA	1	0.2	9	0.61	0.34 (0.04–2.65)	0.3	0.27 (0.03–2.22)	0.224
A allele	52	5.31	183	6.2				

Bold indicates statistical significance (*P* < 0.05)

^a^Adjusted for age, sex, smoking status, alcohol use and BMI status

### Stratified analyses of the association of PPARγ, PPARGC1A, and PPARGC1B SNVs with the risk of GC

To determine whether the effects of these SNVs were modified by factors such as smoking, age, sex, alcohol consumption, and BMI, a stratified analysis was performed(Table 4, Table S1-5). For *PPARγ* rs1801282 C > G SNV, stratified analysis revealed that this variant constituted a GC protective factor in subgroups of age ≥ 61 years, no smoking, and no alcohol consuming (age ≥ 61years: additive model: GC vs. CC: adjusted *P* = 0.043; no smoking: additive model: GC vs. CC: adjusted *P* = 0.033, dominant model: GC + GG vs. CC: adjusted *P* = 0.032; no alcohol consuming: additive model: GC vs. CC: adjusted *P* = 0.006, dominant model: GC + GG vs. CC: adjusted *P* = 0.008; respectively, Table 4).

**Table 4:** Stratified analyses between *PPARγ r*s1801282 C > G polymorphism and GC risk by sex, age, smoking status, alcohol consumption and BMI

Variable	(Case/control)^a^			Adjusted OR^b^ (95% CI); *P*			
	CC	GC	GG	Additive model	Homozygote model	Dominant model	Recessive model
Sex							
Male	301/892	26/103	1/3	0.68 (0.43–1.08) *P*: 0.102	1.07 (0.10–11.44) *P*: 0.959	0.69 (0.44–1.09) *P*: 0.109	1.10 (0.10–11.81) *P*: 0.938
Female	151/425	8/48	0/1	0.47 (0.21–1.04) *P*: 0.061	*– P*: 0.989	0.47 (0.21–1.03) *P*: 0.058	*– P*: 0.990
Age							
< 61	206/622	14/59	0/2	0.66 (0.35–1.24) *P*: 0.196	*– P*: 0.981	0.63 (0.34–1.19) *P*: 0.155	– *P*: 0.981
≥ 61	246/695	20/92	1/2	0.59 (0.35–0.98) ***P***: **0.043**	1.93 (0.17–22.23) *P*: 0.597	0.61 (0.37–1.01) *P*: 0.055	2.02 (0.18–23.22) *P*: 0.572
Smoking status							
Never	289/944	18/101	1/4	0.33 (0.33–0.95) ***P***: **0.031**	0.81 (0.08–8.10) *P*: 0.861	0.57 (0.34–0.95) ***P***: **0.032**	0.85 (0.09–8.48) *P*: 0.893
Ever	163/373	16/50	0/0	0.72 (0.39–1.33) *P*: 0.288	–	0.72 (0.39–1.33) *P*: 0.288	–
Alcohol consumption							
Never	350/1180	21/133	1/3	0.51 (0.32–0.83) ***P***: **0.006**	1.62 (0.16–16.30) P: 0.682	0.53 (0.33–0.85) **P: 0.008**	1.71 (0.17–17.14) *P*: 0.650
Ever	102/137	13/18	0/1	1.11 (0.51–2.43) *P*: 0.789	– *P*: 0.986	1.03 (0.48–2.23) *P*: 0.941	– *P*: 0.986
BMI (kg/m2)							
< 24	329/677	25/80	1/1	0.65 (0.40–1.04) *P*: 0.071	1.98 (0.11–34.90) *P*: 0.640	0.66 (0.41–1.06) *P*: 0.085	2.06 (0.12–36.28) *P*: 0.622
≥ 24	123/640	9/71	0/3	0.57 (0.28–1.19) *P*: 0.137	– *P*: 0.984	0.55 (0.27–1.15) *P*: 0.113	– *P*: 0.984

Bold indicates statistical significance (*P* < 0.05)

^a^The genotyping was successful in 487 (99.39%) gastric cancer cases, and 1472 (99.73%) controls for *PPARγ r*s1801282 C > G

^b^Adjusted for age, sex, BMI, smoking status, alcohol use and BMI (besides stratified factors accordingly) in a logistic regression model

For *PPARγ* rs3856806 C > T SNV, stratified analysis revealed that this variant constituted a GC risk factor in females (additive model: CT vs. CC: adjusted *P* = 0.037; dominant model: CT + TT vs. CC: adjusted *P* = 0.038, Additional file 1: Supplementary Table S1).

For the *PPARGC1A* rs8192678 TT genotype, stratified analysis revealed that this variant constituted a GC protective factor in males. (homozygote model: TT vs. CC: adjusted *P* = 0.045, Additional file 2: Supplementary Table S2)

For *PPARGC1A*rs2970847 C > T SNV, stratified analysis revealed that this variant constituted a GC protective factor in subgroups of BMI ≥ 24 kg/m^2^. (additive model: CT vs. CC: adjusted *P* = 0.028, Additional file 3: Supplementary Table S3)

For *PPARGC1B* rs7732671 G > C SNV, stratified analysis revealed that this variant constituted a GC risk factor in subgroups of BMI ≥ 24 kg/m^2^. (additive model: GC vs. GG: adjusted *P* = 0.027; dominant model: GC + CC vs. GG: adjusted *P* = 0.033, Additional file 4: Supplementary Table S4)

For *PPARGC1B* rs17572019 G > A SNV, stratified analysis revealed that this variant constituted a GC risk factor in subgroups of BMI ≥ 24 kg/m^2^. (additive model: GA vs. GG: adjusted *P* = 0.028; dominant model: GA + AA vs. GG: adjusted *P* = 0.034, Additional file 5: Supplementary Table S5)

## Discussion

This study revealed that *PPARγ* rs1801282 C > G SNV was associated with a decreased risk for GC. In addition, *PPARγ *rs1801282 C > G SNV conferred decreased risk for GC patients among subgroups of age ≥ 61 years, no smoking and no alcohol consuming; *PPARγ *rs3856806 C > T SNV conferred risk for GC patients in females; *PPARGC1A* rs8192678 TT genotype conferred decreased risk for GC patients in male; *PPARGC1A *rs2970847 C > T conferred decreased risk for GC patients in a subgroup of BMI ≥ 24 kg/m^2^; both *PPARGC1B* rs7732671 G > C and *PPARGC1B* rs17572019 G > A conferred risk for GC patients in a subgroup of BMI ≥ 24 kg/m^2^.

The rs1801282 SNV has been reported to be associated with susceptibility to a variety of tumors. However, in some studies such as on breast and colorectal cancers, the correlation tended to vary by race and/or cancer type [34, 36–42]. Several studies focused on the association of rs1801282 SNV with the susceptibility of GC. An Iranian population-based study found that the rs1801282 SNV G allele increased the risk of non-cardia gastric cancer in people with *H. pylori* infection [43]. A Turkish-based study found that rs1801282 C > G SNV not only has an increased risk of GC but is also associated with poor differentiation and metastatic disease in GC [44]. Our study was based on eastern Chinese population, suggesting that rs1801282 SNV is a protective factor for GC, especially in the subgroup aged ≥ 61 years, non-smokers, and no alcohol consuming.

The impact of PPARγ on cancers is complex and bidirectional, PPARγ acts as a tumor promoter by induction of lipogenic gene ACLY, MIG12, FASN, and NR1F1, stem cell-related gene KLF4, ALDH and upregulation Nox1 ROS, and VEGF. PPARγ act as a tumor suppressor by inducing apoptosis through the upregulation of PTEN, suppression of NF-κB, and many other signaling pathways [7, 45, 46]. PPARγ mainly plays an anti-tumorigenesis role in GC. PPARγ expression was low in normal gastric mucosa and significantly higher in GC tissues and was an independent prognostic factor for GC [15, 47] . Enhanced PPARγ activity reduced GC cell migration, invasion, and EMT through upregulation of galectin-9 [47]. Increased expression of PPARγ may reduce proliferation and metastatic potential in GC by inhibiting TERT and ENAH through the Wnt/β-Catenin signaling pathway [48, 49].

Variants in the PPARγ affect gene transcription and expression, which has been intensively studied in metabolic diseases. An in vitro experiment demonstrated that rs1801282 SNV reduced the binding affinity of the receptor to the reactant and induced a reduction in transcriptional activity and was associated with lower BMI and improved insulin sensitivity [50, 51]. However, in a study on obesity, it was observed that the expression of PPARγ2 in subcutaneous fat was higher in heterozygote CG carriers than in the CC genotype [52]. Similar result was observed in another study on metabolic diseases, in which the PPARγ2 mRNA expression level in adipose tissue was higher in rs1801282 GG genotype carriers than in CC genotype carriers [53]. This supports our study to some exten that the rs1801282 heterozygous CG genotype may play an anti-tumorigenesis role in gastric cancer through overexpression of PPARγ2. However, studies on the function of rs1801282 SNV in GC are still lacking. A recent study examined PPARγ rs1801282 SNV in multiple human cancer cell lines, and a heterozygous CG genotype was detected in AGS and Caki-1 cancer cell lines. PPARγ mRNA expression in these cell lines was found to be lower than in cell lines with wild-type such as MCF10A, SK-BR-3, and MDA-MB-468 [54]. Therefore, the impact of rs1801282 SNV on the expression of the *PPARγ* may vary by tumor type and race. Further functional studies are needed to clarify the impact of rs1801282 SNV on GC.

Rs3856806 C > T variant, in exon 6, is a synonymous variant. It is an exon splice enhancement site that may reduce transcription of PPARγ [55]. There is a controversy between rs3856806 C > T variant and cancer susceptibility. In a study, the heterozygous CT genotype of rs3856806 was found to be protective against colorectal cancer, however, in contrast, studies by Jiang et al. and Lin et al. showed an increased risk of colorectal cancer with this genotype [31, 56, 57]. Similarly, several studies have been conducted on breast cancer susceptibility, but the results remain debated [38, 58, 59] . Recently, two meta-analyses reported that the *PPARγ* rs3856806 C > T variant increased overall cancer susceptibility [60, 61]. In a recent study of GC, rs3856806 SNV was found not related to cancer risk [62]. Our results suggested that *PPARγ* rs3856806 C > T constituted a risk factor for GC in women.

In GC tissues and GC cell lines, PPARGC1A expression was upregulated and associated with metastasis, invasion, and induce apoptosis of GC cells [63].  Rs8192678 SNV is the most well-studied variant of the *PPARC1A*, which substitutes glycine with serine at amino acid position 482 in exon 8. Research has also shown that the expression of PPARGC1A is significantly lower in those carrying the minor allele [64]. A study of prostate cancer showed no effect of rs8192678 SNV on cancer susceptibility [65]. In previous studies, the rs8192678 CT genotype represented a protective factor against colorectal cancer, and the rs8192678 TT genotype reduced esophageal squamous carcinoma risk [31, 33]. The present study showed that the rs8192678 TT genotype reduced GC susceptibility in men in a homozygote model. A study based on Spanish population showed that the rs8192678 C > T variant affects insulin sensitivity through a genotype-sex interaction. Men with T allele carriers have lower insulin sensitivity [66]. Since metabolic abnormalities are definite risk factors for gastric cancer, the rs8192678 SNV may affect the development of GC through a similar genotype-sex interaction. Relevant studies and evidence are still lacking and further research is needed.

*PPARGC1A* rs2970847 is a synonymous variant, which is reported associated with the risk of type 2 diabetes, obesity, and insulin resistance [67, 68]. In the previous studies, no association was found between *PPARGC1A* rs2970847 SNV and susceptibility of esophageal, colorectal, and hepatocellular carcinomas [31–33], however, the present study showed that it was a protective factor for GC in the group of BMI ≥ 24 kg/m^2^ in an Additive model. A study of an Iranian population revealed that rs2970847 SNV downregulates insulin signaling pathways and is associated with insulin resistance. Carriers of the T allele of rs2970847 had decreased performance of PPARGC1A and higher risk for obesity [69]. The correlation of rs2970847 with CG and other cancers is still lacking, and further studies are needed.

*PPARGC1B* is located on chromosome 5 and consists of 1023 amino acids. PPARGC1B is highly similar to PPARGC1A in terms of structure, function, and mechanism [70]. Studies showed that the *PPARGC1B* rs7732671 variant increases breast cancer risk and affects cancer progression [71, 72]. The risk of esophageal squamous carcinoma in the high alcohol intake subgroup was promoted by the *PPARGC1B* rs17572019 G > A SNV [33]. In the current study, both *PPARGC1B* rs7732671 G > C and rs17572019 G > A SNVs were found to be a risk factor for GC in the additive and dominant models in the group with BMI ≥ 24 kg/m^2^. A study on breast cancer found that rs7732671 G > C enhances ERRα and ERRγ signaling and modulates aerobic glycolysis [72].  Since the ERRα signaling pathway was shown to promote GC [29], further studies are needed to clarify whether rs7732671 SNV affects GC development through ERRα or other pathways.

To our knowledge, the present study is the largest sample size study to date exploring the relationship between *PPARγ*, *PPARGC1A*, *PPARGC1B* variants, and GC susceptibility. This study demonstrated that the *PPARγ*, *PPARGC1A*, and *PPARGC1B* SNVs were associated with genetic susceptibility to GC. It could be a potential biomarker in the prevention and screening of GC in the Chinese population. However, there were several limitations in this study. First, *H. pylori* infection status was inaccessible, which affect the further subgroup analysis. Second, since both the GC cases and controls were hospital-based, the potential selection bias might have occurred. Third, many other environmental and personal factors might be associated with the etiology of GC, such as socioeconomic status, literacy, and diet, but were not collected and analyzed adequately in this study.

In conclusion, this study indicates that the *PPARγ* rs1801282 C > G SNV was associated with a decreased risk for GC in eastern Chinese population. Future studies with larger populations, detailed *H. pylori* infection status, and functional studies are needed to further clarify the relationship between these SNVs and GC risk.

## Supplementary information

**Additional file 1.** Supplementary Table S1.

**Additional file 2.** Supplementary Table S2.

**Additional file 3.** Supplementary Table S3.

**Additional file 4.** Supplementary Table S4.

**Additional file 5.** Supplementary Table S5.

**Additional file 6.** Supplementary Primers.

**Additional file 7.** Raw_data.

## Data Availability

The data that support the findings of the present are included in the supplementary information file named Additional file 7: Raw_data. The sequences of the primers are provided in the supplementary information file named Additional file 6: Supplementary_Primers.
