# Supplementary material for: Association between PPARγ, PPARGC1A, and PPARGC1B genetic variants and susceptibility of gastric cancer in an Eastern Chinese population
Source: BMC Med Genomics. 2022 Dec 31;15:274. doi: 10.1186/s12920-022-01428-0 (PMC9805199; doi:10.1186/s12920-022-01428-0)
Supplement: Supplementary file 1 — Additional file 1. Supplementary Table S1. [file 12920_2022_1428_MOESM1_ESM.docx]

**Supplementary Table S1** Stratified analyses between *PPARγ* rs3856806 C>T polymorphism and GC risk by sex, age, smoking status, alcohol consumption and BMI

| Variable | (case/control)^a^ | | |  |  | Adjusted OR^b^ (95% CI); *P* | | | |
| --- | --- | --- | --- | --- | --- | --- | --- | --- | --- |
|  | CC | CT | TT |  |  | Additive model | Homozygote model | Dominant model | Recessive model |
| Sex |  |  |  |  |  |  |  |  |  |
| Male | 193/574 | 122/388 | 13/36 |  |  | 0.93(0.71-1.22)  *P*: 0.592 | 0.93(0.47-1.83)  *P*: 0.825 | 0.93(0.71-1.21)  *P*: 0.580 | 0.95(0.49-1.86) |
|  |  |  |  |  |  |  |  |  | *P*: 0.889 |
| Female | 85/294 | 66/156 | 8/24 |  |  | 1.51(1.03-2.23)  ***P*: 0.037** | 1.31(0.55-3.10)  *P*: 0.539 | 1.49(1.02-2.16)  ***P*: 0.038** | 1.11(0.48-2.59) |
|  |  |  |  |  |  |  |  |  | *P*: 0.802 |
| Age |  |  |  |  |  |  |  |  |  |
| <61 | 130/398 | 82/260 | 8/25 |  |  | 0.99(0.71-1.39)  *P*: 0.972 | 0.92(0.39-2.18)  *P*: .8410 | 0.99(0.71-1.37)  *P*: 0.935 | 0.92(0.39-2.16) |
|  |  |  |  |  |  |  |  |  | *P*: 0.843 |
| ≥61 | 148/470 | 106/284 | 13/35 |  |  | 1.16(0.86-1.56)  *P*: 0.320 | 1.13(0.57-2.21)  *P*: 0.731 | 1.16(0.87-1.54)  *P*: 0.315 | 1.06(0.55-2.07) |
|  |  |  |  |  |  |  |  |  | *P*: 0.861 |
| Smoking status |  |  |  |  |  |  |  |  |  |
| Never | 168/629 | 125/373 | 15/47 |  |  | 1.26(0.96-1.65)  *P*: 0.098 | 1.08(0.58-2.01)  *P*: 0.804 | 1.24(0.95-1.61)  *P*: 0.114 | 0.99(0.54-1.82) |
|  |  |  |  |  |  |  |  |  | *P*: 0.967 |
| Ever | 110/239 | 63/171 | 6/13 |  |  | 0.82(0.56-1.20)  *P*: 0.308 | 0.93(0.33-2.63)  *P*: 0.893 | 0.83(0.57-1.20)  *P*: 0.321 | 1.01(0.36-2.81) |
|  |  |  |  |  |  |  |  |  | *P*: 0.990 |
| Alcohol consumption |  |  |  |  |  |  |  |  |  |
| Never | 213/782 | 145/478 | 14/56 |  |  | 1.14(0.89-1.45)  *P*: 0.311 | 0.85(0.46-1.58)  *P*: 0.614 | 1.10(0.87-1.40)  *P*: 0.418 | 0.81(0.44-1.49) |
|  |  |  |  |  |  |  |  |  | *P*: 0.503 |
| Ever | 65/86 | 43/66 | 7/4 |  |  | 0.91(0.55-1.52)  *P*: 0.720 | 2.20(0.60-8.05) | 0.99(0.60-1.63)  *P*: 0.963 | 2.29(0.63-8.23) |
|  |  |  |  |  |  |  | *P*: 0.235 |  | *P*: 0.206 |
| BMI(kg/m^2^) |  |  |  |  |  |  |  |  |  |
| < 24 | 200/452 | 140/269 | 15/37 |  |  | 1.18(0.90-1.54)  *P*: 0.235 | 0.93(0.50-1.76)  *P*: 0.831 | 1.15(0.89-1.49)  *P*: 0.299 | 0.88(0.47-1.64) |
|  |  |  |  |  |  |  |  |  | *P*: 0.676 |
| ≥ 24 | 78/416 | 48/275 | 6/23 |  |  | 0.91(0.61-1.35)  *P*: 0.623 | 1.44(0.56-3.70)  *P*: 0.450 | 0.95(0.65-1.39)  *P*: 0.774 | 1.49(0.59-3.79) |
|  |  |  |  |  |  |  |  |  | *P*: 0.399 |

^a^The genotyping was successful in 487 (99.39%) gastric cancer cases, and 1472 (99.73%) controls for *PPARγ* rs3856806 C>T.

^b^Adjusted for age, sex, BMI, smoking status, alcohol use and BMI (besides stratified factors accordingly) in a logistic regression model.
