# Supplementary material for: Association between PPARγ, PPARGC1A, and PPARGC1B genetic variants and susceptibility of gastric cancer in an Eastern Chinese population
Source: BMC Med Genomics. 2022 Dec 31;15:274. doi: 10.1186/s12920-022-01428-0 (PMC9805199; doi:10.1186/s12920-022-01428-0)
Supplement: Supplementary file 2 — Additional file 2. Supplementary Table S2. [file 12920_2022_1428_MOESM2_ESM.docx]

**Supplementary Table S2** Stratified analyses between *PPARGC1A* rs8192678 C>T polymorphism and GC risk by sex, age, smoking status, alcohol consumption and BMI

| Variable | (case/control)^a^ | | |  |  | Adjusted OR^b^ (95% CI); *P* | | | |
| --- | --- | --- | --- | --- | --- | --- | --- | --- | --- |
|  | CC | CT | TT |  |  | Additive model | Homozygote model | Dominant model | Recessive model |
| Sex |  |  |  |  |  |  |  |  |  |
| Male | 121/307 | 153/485 | 54/206 |  |  | 0.81(0.61-1.08)  *P*: 0.148 | 0.68(0.46-0.99)  ***P*: 0.045** | 0.77(0.59-1.01)  *P*: 0.059 | 0.77(0.55-1.08)  *P*: 0.130 |
| Female | 48/147 | 83/241 | 28/86 |  |  | 1.16(0.76-1.77)  *P*: 0.500 | 1.14(0.65-1.98)  *P*: 0.651 | 1.15(0.77-1.72)  *P*: 0.493 | 1.04(0.64-1.68)  *P*: 0.883 |
| Age |  |  |  |  |  |  |  |  |  |
| <61 | 77/216 | 105/339 | 38/128 |  |  | 0.86(0.60-1.23)  *P*: 0.403 | 0.92(0.58-1.48)  *P*: 0.738 | 0.87(0.62-1.23)  *P*: 0.439 | 1.01(0.66-1.54)  *P*: 0.959 |
| ≥61 | 92/238 | 131/387 | 44/164 |  |  | 0.93(0.68-1.28)  *P*: 0.653 | 0.72(0.48-1.10)  *P*: 0.130 | 0.87(0.64-1.17)  *P*: 0.356 | 0.76(0.52-1.10)  *P*: 0.141 |
| Smoking status |  |  |  |  |  |  |  |  |  |
| Never | 106/320 | 153/522 | 49/207 |  |  | 0.90(0.67-1.20)  *P*: 0.479 | 0.75(0.51-1.10)  *P*: 0.137 | 0.86(0.65-1.13)  *P*: 0.270 | 0.79(0.56-1.12)  *P*: 0.192 |
| Ever | 63/134 | 83/204 | 33/85 |  |  | 0.88(0.58-1.32)  *P*: 0.532 | 0.87(0.51-1.46)  *P*: 0.591 | 0.87(0.59-1.29)  *P*: 0.494 | 0.94(0.59-1.49)  *P*: 0.780 |
| Alcohol consumption |  |  |  |  |  |  |  |  |  |
| Never | 127/410 | 184/644 | 61/262 |  |  | 0.94(0.72-1.22)  *P*: 0.649 | 0.77(0.54-1.08)  *P*: 0.132 | 0.89(0.69-1.14)  *P*: 0.358 | 0.79(0.58-1.08)  *P*: 0.146 |
| Ever | 42/44 | 52/82 | 21/30 |  |  | 0.68(0.39-1.19)  *P*: 0.180 | 0.86(0.42-1.77) | 0.73(0.43-1.23)  *P*: 0.233 | 1.08(0.57-2.05) |
|  |  |  |  |  |  |  | *P*: 0.675 |  | *P*: 0.821 |
| BMI(kg/m^2^) |  |  |  |  |  |  |  |  |  |
| < 24 | 128/244 | 168/368 | 59/146 |  |  | 0.86(0.65-1.15)  *P*: 0.316 | 0.79(0.55-1.16)  *P*: 0.230 | 0.84(0.65-1.11)  *P*: 0.218 | 0.87(0.62-1.21)  *P*: 0.400 |
| ≥ 24 | 41/210 | 68/358 | 23/146 |  |  | 0.94(0.61-1.44)  *P*: 0.777 | 0.74(0.42-1.29)  *P*: 0.283 | 0.88(0.59-1.32)  *P*: 0.537 | 0.76(0.47-1.25)  *P*: 0.285 |

^a^The genotyping was successful in 487 (99.39%) gastric cancer cases, and 1472 (99.73%) controls for *PPARGC1A* rs8192678 C>T.

^b^Adjusted for age, sex, BMI, smoking status, alcohol use and BMI (besides stratified factors accordingly) in a logistic regression model.
