# Supplementary material for: Association between PPARγ, PPARGC1A, and PPARGC1B genetic variants and susceptibility of gastric cancer in an Eastern Chinese population
Source: BMC Med Genomics. 2022 Dec 31;15:274. doi: 10.1186/s12920-022-01428-0 (PMC9805199; doi:10.1186/s12920-022-01428-0)
Supplement: Supplementary file 3 — Additional file 3. Supplementary Table S3. [file 12920_2022_1428_MOESM3_ESM.docx]

**Supplementary Table S3** Stratified analyses between *PPARGC1A* rs2970847 C>T polymorphism and GC risk by sex, age, smoking status, alcohol consumption and BMI

| Variable | (case/control)^a^ | | |  |  | Adjusted OR^b^ (95% CI); *P* | | | |
| --- | --- | --- | --- | --- | --- | --- | --- | --- | --- |
|  | CC | CT | TT |  |  | Additive model | Homozygote model | Dominant model | Recessive model |
| Sex |  |  |  |  |  |  |  |  |  |
| Male | 202/605 | 110/345 | 16/48 |  |  | 0.97(0.73-1.28)  *P*: 0.827 | 1.15(0.62-2.12)  *P*: 0.661 | 0.99(0.76-1.29)  *P*: 0.937 | 1.16(0.63-2.12)  *P*: 0.632 |
| Female | 101/285 | 50/170 | 8/19 |  |  | 0.76(0.51-1.13)  *P*: 0.177 | 1.17(0.48-2.83)  *P*: 0.734 | 0.80(0.54-1.17)  *P*: 0.248 | 1.29(0.54-3.09)  *P*: 0.572 |
| Age |  |  |  |  |  |  |  |  |  |
| <61 | 131/429 | 79/216 | 10/38 |  |  | 1.07(0.76-1.50)  *P*: 0.721 | 0.90(0.42-1.94)  *P*: 0.789 | 1.04(0.75-1.45)  *P*: 0.805 | 0.88(0.41-1.88)  *P*: 0.742 |
| ≥61 | 172/461 | 81/299 | 14/29 |  |  | 0.75(0.55-1.02)  *P*: 0.070 | 1.42(0.72-2.79)  *P*: 0.310 | 0.81(0.60-1.09)  *P*: 0.156 | 1.57(0.81-3.07)  *P*: 0.184 |
| Smoking status |  |  |  |  |  |  |  |  |  |
| Never | 192/624 | 99/376 | 17/49 |  |  | 0.84(0.63-1.11)  *P*: 0.214 | 1.24(0.69-2.23)  *P*: 0.472 | 0.88(0.67-1.15)  *P*: 0.350 | 1.32(0.74-2.36)  *P*: 0.346 |
| Ever | 111/266 | 61/139 | 7/18 |  |  | 1.06(0.71-1.56)  *P*: 0.786 | 0.97(0.37-2.53)  *P*: 0.953 | 1.05(0.72-1.53)  *P*: 0.814 | 0.95(0.37-2.45)  *P*: 0.922 |
| Alcohol consumption |  |  |  |  |  |  |  |  |  |
| Never | 230/794 | 123/461 | 19/61 |  |  | 0.91(0.71-1.17)  *P*: 0.471 | 1.18(0.68-2.03)  *P*: 0.564 | 0.94(0.74-1.20)  *P*: 0.617 | 1.21(0.71-2.09)  *P*: 0.481 |
| Ever | 73/96 | 37/54 | 5/6 |  |  | 0.84(0.49-1.42)  *P*: 0.509 | 1.04(0.30-3.68) | 0.86(0.51-1.43)  *P*: 0.553 | 1.11(0.32-3.86) |
|  |  |  |  |  |  |  | *P*: 0.948 |  | *P*: 0.868 |
| BMI(kg/m^2^) |  |  |  |  |  |  |  |  |  |
| < 24 | 214/464 | 127/262 | 14/32 |  |  | 1.04(0.79-1.36)  *P*: 0.797 | 0.95(0.49-1.85)  *P*: 0.888 | 1.03(0.79-1.34)  *P*: 0.840 | 0.94(0.49-1.81)  *P*: 0.856 |
| ≥ 24 | 89/426 | 33/253 | 10/35 |  |  | 0.61(0.40-0.95)  ***P*: 0.028** | 1.46(0.69-3.09)  *P*: 0.324 | 0.71(0.48-1.06)  *P*: 0.096 | 1.71(0.81-3.57)  *P*: 0.157 |

^a^The genotyping was successful in 487 (99.39%) gastric cancer cases, and 1472 (99.73%) controls for *PPARGC1A* rs2970847 C>T.

^b^Adjusted for age, sex, BMI, smoking status, alcohol use and BMI (besides stratified factors accordingly) in a logistic regression model.
