# Supplementary material for: Association between PPARγ, PPARGC1A, and PPARGC1B genetic variants and susceptibility of gastric cancer in an Eastern Chinese population
Source: BMC Med Genomics. 2022 Dec 31;15:274. doi: 10.1186/s12920-022-01428-0 (PMC9805199; doi:10.1186/s12920-022-01428-0)
Supplement: Supplementary file 4 — Additional file 4. Supplementary Table S4. [file 12920_2022_1428_MOESM4_ESM.docx]

**Supplementary Table S4** Stratified analyses between *PPARGC1B* rs7732671 G>C polymorphism and GC risk by sex, age, smoking status, alcohol consumption and BMI

| Variable | (case/control)^a^ | | |  |  | Adjusted OR^b^ (95% CI); *P* | | | |
| --- | --- | --- | --- | --- | --- | --- | --- | --- | --- |
|  | GG | GC | CC |  |  | Additive model | Homozygote model | Dominant model | Recessive model |
| Sex |  |  |  |  |  |  |  |  |  |
| Male | 297/885 | 30/110 | 1/3 |  |  | 0.87(0.56-1.34)  *P*: 0.519 | 1.24(0.13-12.25)  *P*: 0.853 | 0.88(0.57-1.35)  *P*: 0.544 | 1.26(0.13-12.44)  *P*: 0.843 |
| Female | 139/414 | 20/56 | 0/4 |  |  | 1.15(0.65-2.01)  *P*: 0.639 | -  *P*: 0.984 | 1.05(0.60-1.83)  *P*: 0.870 | -  *P*: 0.984 |
| Age |  |  |  |  |  |  |  |  |  |
| <61 | 193/597 | 26/82 | 1/4 |  |  | 1.05(0.64-1.72)  *P*: 0.852 | 0.73(0.08-6.95)  *P*: 0.784 | 1.03(0.63-1.68)  *P*: 0.902 | 0.73(0.08-6.91)  *P*: 0.780 |
| ≥61 | 243/702 | 24/84 | 0/3 |  |  | 0.87(0.54-1.42)  *P*: 0.578 | -  *P*: 0.980 | 0.84(0.52-1.37)  *P*: 0.485 | -  *P*: 0.980 |
| Smoking status |  |  |  |  |  |  |  |  |  |
| Never | 271/923 | 36/120 | 1/6 |  |  | 1.10(0.74-1.66)  *P*: 0.634 | 0.47(0.06-3.93)  *P*: 0.483 | 1.07(0.72-1.59)  *P*: 0.755 | 0.46(0.06-3.89)  *P*: 0.477 |
| Ever | 165/376 | 14/46 | 0/1 |  |  | 0.68(0.36-1.30)  *P*: 0.244 | -  *P*: 0.989 | 0.67(0.35-1.29)  *P*: 0.232 | -  *P*: 0.989 |
| Alcohol consumption |  |  |  |  |  |  |  |  |  |
| Never | 333/1155 | 38/154 | 1/7 |  |  | 0.89(0.61-1.30)  *P*: 0.544 | 0.42(0.05-3.47)  *P*: 0.421 | 0.86(0.59-1.26)  *P*: 0.449 | 0.43(0.05-3.52)  *P*: 0.428 |
| Ever | 103/144 | 12/12 | 0/0 |  |  | 1.40(0.59-3.31)  *P*: 0.443 | - | 1.40(0.59-3.31)  *P*: 0.443 | - |
|  |  |  |  |  |  |  |  |  |  |
| BMI(kg/m^2^) |  |  |  |  |  |  |  |  |  |
| < 24 | 327/667 | 27/86 | 1/5 |  |  | 0.65(0.41-1.03)  *P*: 0.067 | 0.47(0.05-4.09)  *P*: 0.495 | 0.64(0.41-1.01)  *P*: 0.054 | 0.49(0.06-4.27)  *P*: 0.519 |
| ≥ 24 | 109/632 | 23/80 | 0/2 |  |  | 1.79(1.07-3.00)  ***P*: 0.027** | -  *P*: 0.987 | 1.75(1.05-2.93)  ***P*: 0.033** | -  *P*: 0.987 |

^a^The genotyping was successful in 487 (99.39%) gastric cancer cases, and 1472 (99.73%) controls for *PPARGC1B* rs7732671 G>C.

^b^Adjusted for age, sex, BMI, smoking status, alcohol use and BMI (besides stratified factors accordingly) in a logistic regression model.
