# Supplementary material for: Association between PPARγ, PPARGC1A, and PPARGC1B genetic variants and susceptibility of gastric cancer in an Eastern Chinese population
Source: BMC Med Genomics. 2022 Dec 31;15:274. doi: 10.1186/s12920-022-01428-0 (PMC9805199; doi:10.1186/s12920-022-01428-0)
Supplement: Supplementary file 5 — Additional file 5. Supplementary Table S5. [file 12920_2022_1428_MOESM5_ESM.docx]

**Supplementary Table S5** Stratified analyses between *PPARGC1B* rs17572019 G>A polymorphism and GC risk by sex, age, smoking status, alcohol consumption and BMI

| Variable | (case/control)^a^ | | |  |  | Adjusted OR^b^ (95% CI); *P* | | | |
| --- | --- | --- | --- | --- | --- | --- | --- | --- | --- |
|  | GG | GA | AA |  |  | Additive model | Homozygote model | Dominant model | Recessive model |
| Sex |  |  |  |  |  |  |  |  |  |
| Male | 296/884 | 30/109 | 1/5 |  |  | 0.89(0.58-1.39)  *P*: 0.615 | 0.51(0.06-4.70)  *P*: 0.553 | 0.87(0.57-1.35)  *P*: 0.540 | 0.52(0.06-4.75)  *P*: 0.559 |
| Female | 139/414 | 20/56 | 0/4 |  |  | 1.15(0.65-2.01)  *P*: 0.639 | -  *P*: 0.984 | 1.05(0.60-1.83)  *P*: 0.870 | -  *P*: 0.984 |
| Age |  |  |  |  |  |  |  |  |  |
| <61 | 193/596 | 26/82 | 1/5 |  |  | 1.06(0.65-1.74)  *P*: 0.817 | 0.57(0.06-5.07)  *P*: 0.611 | 1.03(0.63-1.67)  *P*: 0.917 | 0.56(0.06-5.04)  *P*: 0.607 |
| ≥61 | 242/702 | 24/83 | 0/4 |  |  | 0.90(0.55-1.46)  *P*: 0.660 | -  *P*: 0.976 | 0.85(0.52-1.37)  *P*: 0.494 | -  *P*: 0.976 |
| Smoking status |  |  |  |  |  |  |  |  |  |
| Never | 271/922 | 36/121 | 1/6 |  |  | 1.10(0.73-1.65)  *P*: 0.653 | 0.47(0.06-3.93)  *P*: 0.483 | 1.06(0.71-1.58)  *P*: 0.775 | 0.46(0.06-3.89)  *P*: 0.477 |
| Ever | 164/376 | 14/44 | 0/3 |  |  | 0.73(0.38-1.41)  *P*: 0.349 | -  *P*: 0.985 | 0.68(0.35-1.30)  *P*: 0.239 | -  *P*: 0.985 |
| Alcohol consumption |  |  |  |  |  |  |  |  |  |
| Never | 332/1154 | 38/154 | 1/8 |  |  | 0.90(0.61-1.31)  *P*: 0.572 | 0.36(0.04-2.93)  *P*: 0.340 | 0.86(0.59-1.26)  *P*: 0.446 | 0.37(0.05-2.96)  *P*: 0.345 |
| Ever | 103/144 | 12/11 | 0/1 |  |  | 1.55(0.64-3.74)  *P*: 0.328 | - | 1.40(0.59-3.31)  *P*: 0.443 | - |
|  |  |  |  |  |  |  | *P*: 0.991 |  | *P*: 0.986 |
| BMI(kg/m^2^) |  |  |  |  |  |  |  |  |  |
| < 24 | 327/667 | 27/84 | 1/7 |  |  | 0.67(0.43-1.07)  *P*: 0.091 | 0.29(0.04-2.40)  *P*: 0.251 | 0.64(0.41-1.01)  *P*: 0.054 | 0.30(0.04-2.49)  *P*: 0.265 |
| ≥ 24 | 108/631 | 23/81 | 0/2 |  |  | 1.79(1.1.07-2.99)  ***P*: 0.028** | -  *P*: 0.987 | 1.74(1.04-2.92)  ***P*: 0.034** | -  *P*: 0.987 |

^a^The genotyping was successful in 486 (99.18%) gastric cancer cases, and 1472 (99.73%) controls for *PPARGC1B* rs17572019 G>A.

^b^Adjusted for age, sex, BMI, smoking status, alcohol use and BMI (besides stratified factors accordingly) in a logistic regression model.
