# Supplementary material for: Association between PPARγ, PPARGC1A, and PPARGC1B genetic variants and susceptibility of gastric cancer in an Eastern Chinese population
Source: BMC Med Genomics. 2022 Dec 31;15:274. doi: 10.1186/s12920-022-01428-0 (PMC9805199; doi:10.1186/s12920-022-01428-0)
Supplement: Supplementary file 6 — Additional file 6. Supplementary Primers. [file 12920_2022_1428_MOESM6_ESM.docx]

**Primers**

| SNP |  | Primer | Primer Sequences | Length |
| --- | --- | --- | --- | --- |
| rs17572019 | A1 | rs17572019_GF | GGCAGACCCCGGTGCCCAGG | 20 |
|  | A2 | rs17572019_AF | GGCAGACCCCGGTGCCCAGA | 20 |
|  | PC | rs17572019_3F | TTTCCCAGGAAGACATGCAGG | 21 |
| rs7732671 | A1 | rs7732671_GF | GGACAGCACCCAAGACAAGATGG | 23 |
|  | A2 | rs7732671_CF | GGACAGCACCCAAGACAAGATGC | 23 |
|  | PC | rs7732671_3F | CTCCCATGATGCAGTCTCAGAGC | 23 |
| rs8192678 | A1 | rs8192678_CR | GACGACGAAGCAGACAAGAACG | 22 |
|  | A2 | rs8192678_TR | GACGACGAAGCAGACAAGAGCA | 22 |
|  | PC | rs8192678_3R | GTGAACTGAGGGACAGTGATTTCAG | 25 |
| rs3856806 | A1 | rs3856806_CR | TTGATCACCTGCAGTAGCTGCTCG | 24 |
|  | A2 | rs3856806_TR | TTGATCACCTGCAGTAGCTGCCCA | 24 |
|  | PC | rs3856806_3R | TGTTCCGTGACAATCTGTCTGAGG | 24 |
| rs1801282 | A1 | rs1801282_CR | AGTGTATCAGTGAAGGAATCGCTTTCAGG | 29 |
|  | A2 | rs1801282_GR | AGTGTATCAGTGAAGGAATCGCTTTCAGC | 29 |
|  | PC | rs1801282_3R | GTCAATAGGAGAATCTCCCAGAGTTTCA | 28 |
| rs2970847 | A1 | rs2970847_TR | ATGACTATTGCCAGTCAATTAATTCCAAACCA | 32 |
|  | A2 | rs2970847_CR | ATGACTATTGCCAGTCAATTAATTCCAAACCG | 32 |
|  | PC | rs2970847_3R | GAAATACTCATTAATATATCACAGGAGCTCCA | 32 |
